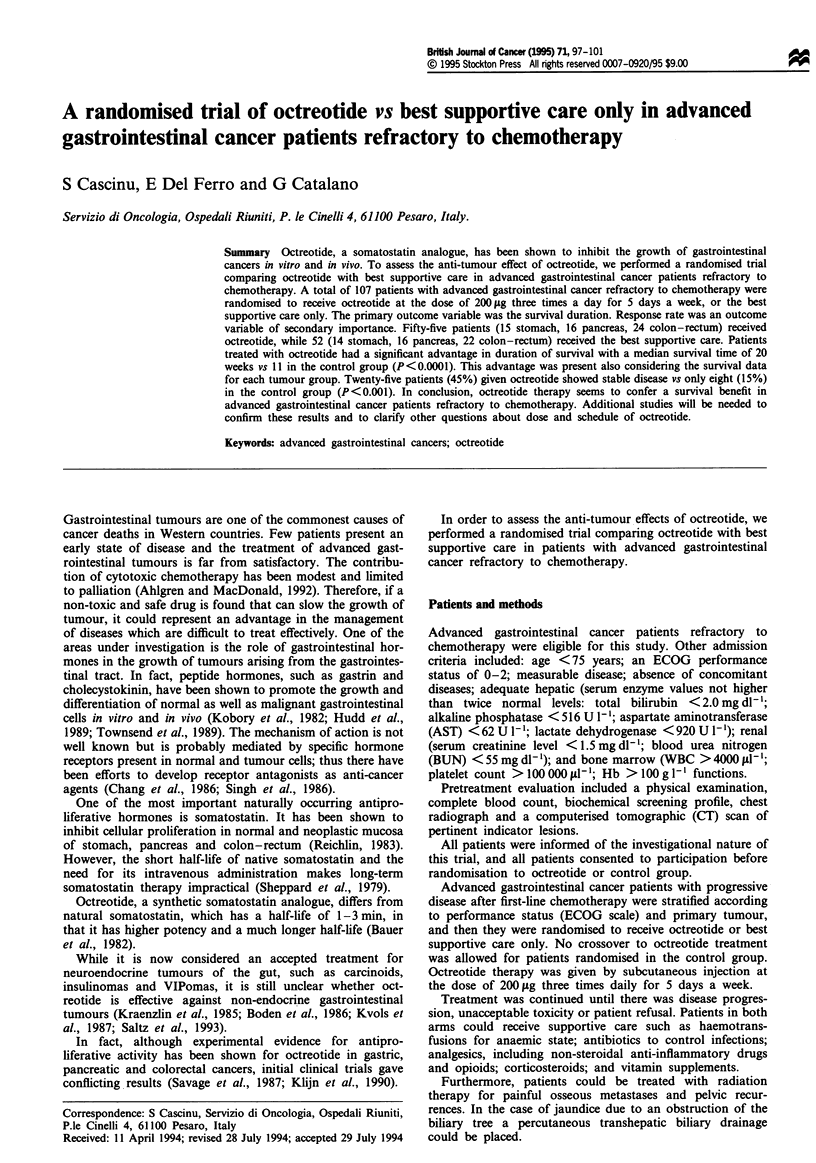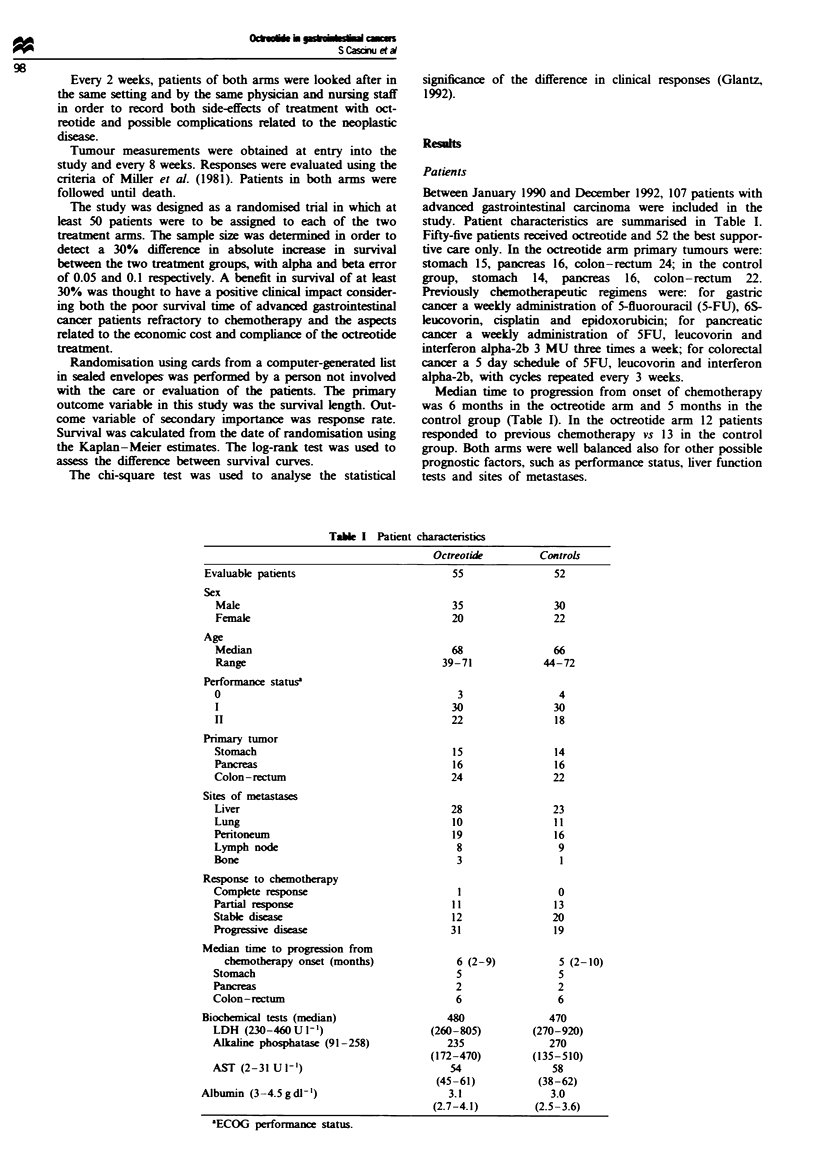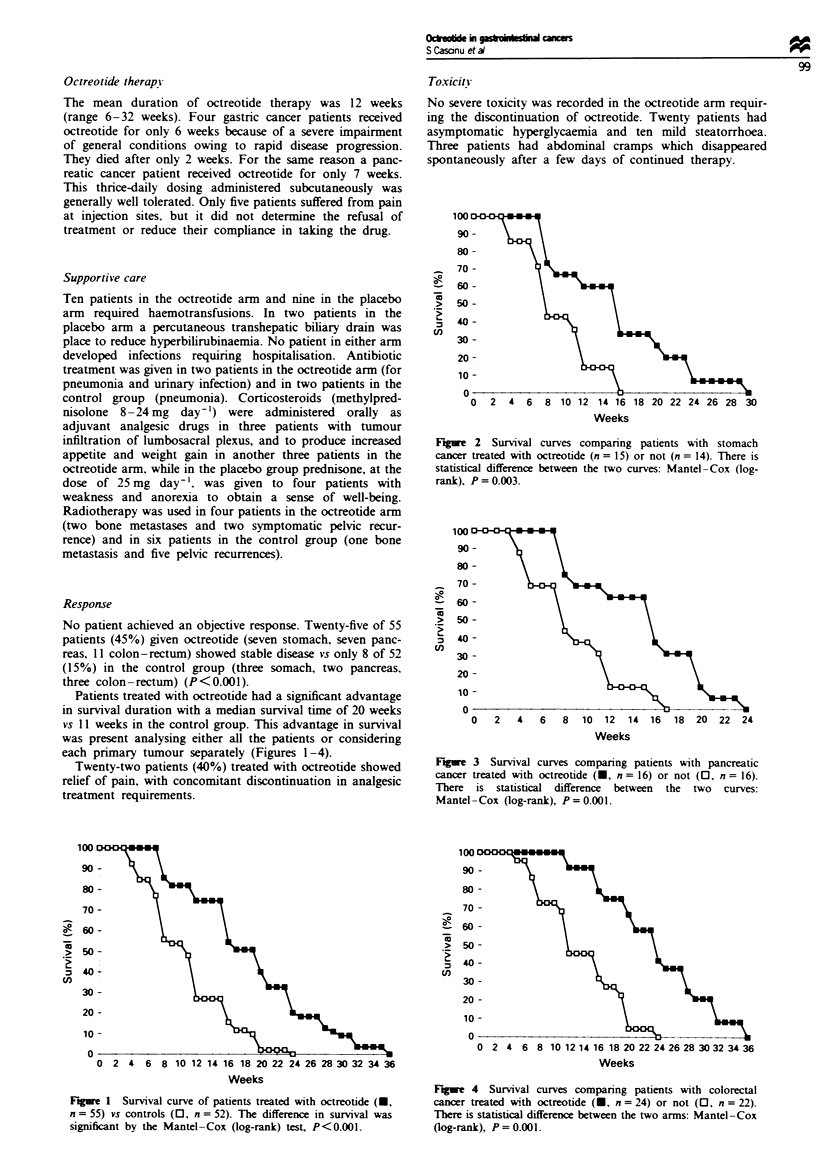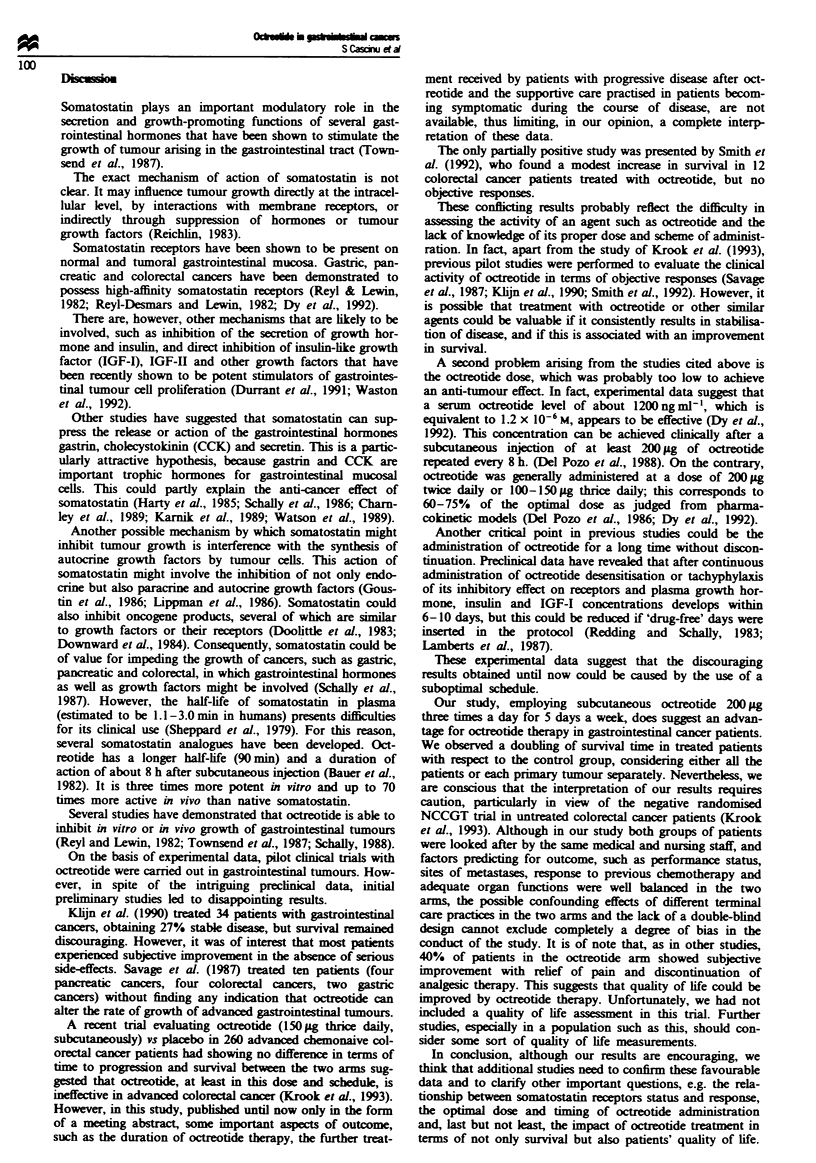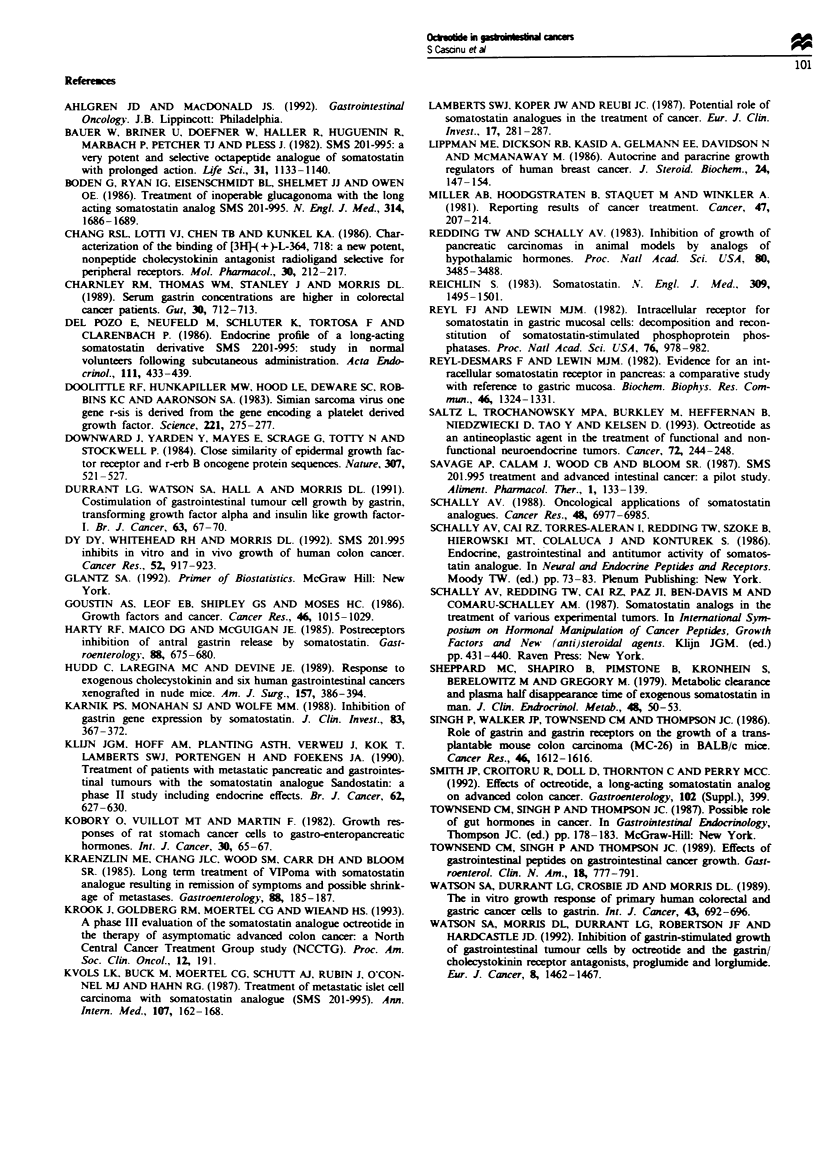# A randomised trial of octreotide vs best supportive care only in advanced gastrointestinal cancer patients refractory to chemotherapy.

**DOI:** 10.1038/bjc.1995.19

**Published:** 1995-01

**Authors:** S. Cascinu, E. Del Ferro, G. Catalano

**Affiliations:** Servizio di Oncologia, Ospedali Riuniti, Pesaro, Italy.

## Abstract

Octreotide, a somatostatin analogue, has been shown to inhibit the growth of gastrointestinal cancers in vitro and in vivo. To assess the anti-tumour effect of octreotide, we performed a randomised trial comparing octreotide with best supportive care in advanced gastrointestinal cancer patients refractory to chemotherapy. A total of 107 patients with advanced gastrointestinal cancer refractory to chemotherapy were randomised to receive octreotide at the dose of 200 micrograms three times a day for 5 days a week, or the best supportive care only. The primary outcome variable was the survival duration. Response rate was an outcome variable of secondary importance. Fifty-five patients (15 stomach, 16 pancreas, 24 colon-rectum) received octreotide, while 52 (14 stomach, 16 pancreas, 22 colon-rectum) received the best supportive care. Patients treated with octreotide had a significant advantage in duration of survival with a median survival time of 20 weeks vs 11 in the control group (P < 0.0001). This advantage was present also considering the survival data for each tumour group. Twenty-five patients (45%) given octreotide showed stable disease vs only eight (15%) in the control group (P < 0.001). In conclusion, octreotide therapy seems to confer a survival benefit in advanced gastrointestinal cancer patients refractory to chemotherapy. Additional studies will be needed to confirm these results and to clarify other questions about dose and schedule of octreotide.


### A randomised trial of octreotide *vs* best supportive care only in advanced gastrointestinal cancer patients refractory to chemotherapy

S Cascinu, E Del Ferro and G Catalano

*Servizio di Oncologia, Ospedali Riuniti, P. le Cinelli 4, 61100 Pesaro, Italy.*

**Summary** Octreotide, a somatostatin analogue, has been shown to inhibit the growth of gastrointestinal cancers *in vitro* and *in vivo*. To assess the anti-tumour effect of octreotide, we performed a randomised trial comparing octreotide with best supportive care in advanced gastrointestinal cancer patients refractory to chemotherapy. A total of 107 patients with advanced gastrointestinal cancer refractory to chemotherapy were randomised to receive octreotide at the dose of 200 μg three times a day for 5 days a week, or the best supportive care only. The primary outcome variable was the survival duration. Response rate was an outcome variable of secondary importance. Fifty-five patients (15 stomach, 16 pancreas, 24 colon-rectum) received octreotide, while 52 (14 stomach, 16 pancreas, 22 colon-rectum) received the best supportive care. Patients treated with octreotide had a significant advantage in duration of survival with a median survival time of 20 weeks *vs* 11 in the control group ($P<0.0001$). This advantage was present also considering the survival data for each tumour group. Twenty-five patients (45%) given octreotide showed stable disease *vs* only eight (15%) in the control group ($P<0.001$). In conclusion, octreotide therapy seems to confer a survival benefit in advanced gastrointestinal cancer patients refractory to chemotherapy. Additional studies will be needed to confirm these results and to clarify other questions about dose and schedule of octreotide.

**Keywords:** advanced gastrointestinal cancers; octreotide

Gastrointestinal tumours are one of the commonest causes of cancer deaths in Western countries. Few patients present an early state of disease and the treatment of advanced gastrointestinal tumours is far from satisfactory. The contribution of cytotoxic chemotherapy has been modest and limited to palliation (Ahlgren and MacDonald, 1992). Therefore, if a non-toxic and safe drug is found that can slow the growth of tumour, it could represent an advantage in the management of diseases which are difficult to treat effectively. One of the areas under investigation is the role of gastrointestinal hormones in the growth of tumours arising from the gastrointestinal tract. In fact, peptide hormones, such as gastrin and cholecystokinin, have been shown to promote the growth and differentiation of normal as well as malignant gastrointestinal cells *in vitro* and *in vivo* (Kobory *et al.*, 1982; Hudd *et al.*, 1989; Townsend *et al.*, 1989). The mechanism of action is not well known but is probably mediated by specific hormone receptors present in normal and tumour cells; thus there have been efforts to develop receptor antagonists as anti-cancer agents (Chang *et al.*, 1986; Singh *et al.*, 1986).

One of the most important naturally occurring antiproliferative hormones is somatostatin. It has been shown to inhibit cellular proliferation in normal and neoplastic mucosa of stomach, pancreas and colon-rectum (Reichlin, 1983). However, the short half-life of native somatostatin and the need for its intravenous administration makes long-term somatostatin therapy impractical (Sheppard *et al.*, 1979).

Octreotide, a synthetic somatostatin analogue, differs from natural somatostatin, which has a half-life of 1-3 min, in that it has higher potency and a much longer half-life (Bauer *et al.*, 1982).

While it is now considered an accepted treatment for neuroendocrine tumours of the gut, such as carcinoids, insulinomas and VIPomas, it is still unclear whether octreotide is effective against non-endocrine gastrointestinal tumours (Kraenzlin *et al.*, 1985; Boden *et al.*, 1986; Kvols *et al.*, 1987; Saltz *et al.*, 1993).

In fact, although experimental evidence for antiproliferative activity has been shown for octreotide in gastric, pancreatic and colorectal cancers, initial clinical trials gave conflicting results (Savage *et al.*, 1987; Klijn *et al.*, 1990).

Correspondence: S Cascinu, Servizio di Oncologia, Ospedali Riuniti, P.le Cinelli 4, 61100 Pesaro, Italy
Received: 11 April 1994; revised 28 July 1994; accepted 29 July 1994

In order to assess the anti-tumour effects of octreotide, we performed a randomised trial comparing octreotide with best supportive care in patients with advanced gastrointestinal cancer refractory to chemotherapy.

### Patients and methods

Advanced gastrointestinal cancer patients refractory to chemotherapy were eligible for this study. Other admission criteria included: age <75 years; an ECOG performance status of 0-2; measurable disease; absence of concomitant diseases; adequate hepatic (serum enzyme values not higher than twice normal levels: total bilirubin $<2.0\ mg\ dl^{-1}$; alkaline phosphatase $<516\ U\ l^{-1}$; aspartate aminotransferase (AST) $<62\ U\ l^{-1}$; lactate dehydrogenase $<920\ U\ l^{-1}$); renal (serum creatinine level $<1.5\ mg\ dl^{-1}$; blood urea nitrogen (BUN) $<55\ mg\ dl^{-1}$); and bone marrow (WBC $>4000\ \mu l^{-1}$; platelet count $>100\,000\ \mu l^{-1}$; Hb $>100\ g\ l^{-1}$ functions.

Pretreatment evaluation included a physical examination, complete blood count, biochemical screening profile, chest radiograph and a computerised tomographic (CT) scan of pertinent indicator lesions.

All patients were informed of the investigational nature of this trial, and all patients consented to participation before randomisation to octreotide or control group.

Advanced gastrointestinal cancer patients with progressive disease after first-line chemotherapy were stratified according to performance status (ECOG scale) and primary tumour, and then they were randomised to receive octreotide or best supportive care only. No crossover to octreotide treatment was allowed for patients randomised in the control group. Octreotide therapy was given by subcutaneous injection at the dose of 200 μg three times daily for 5 days a week.

Treatment was continued until there was disease progression, unacceptable toxicity or patient refusal. Patients in both arms could receive supportive care such as haemotransfusions for anaemic state; antibiotics to control infections; analgesics, including non-steroidal anti-inflammatory drugs and opioids; corticosteroids; and vitamin supplements.

Furthermore, patients could be treated with radiation therapy for painful osseous metastases and pelvic recurrences. In the case of jaundice due to an obstruction of the biliary tree a percutaneous transhepatic biliary drainage could be placed.

Every 2 weeks, patients of both arms were looked after in the same setting and by the same physician and nursing staff in order to record both side-effects of treatment with octreotide and possible complications related to the neoplastic disease.

Tumour measurements were obtained at entry into the study and every 8 weeks. Responses were evaluated using the criteria of Miller *et al.* (1981). Patients in both arms were followed until death.

The study was designed as a randomised trial in which at least 50 patients were to be assigned to each of the two treatment arms. The sample size was determined in order to detect a 30% difference in absolute increase in survival between the two treatment groups, with alpha and beta error of 0.05 and 0.1 respectively. A benefit in survival of at least 30% was thought to have a positive clinical impact considering both the poor survival time of advanced gastrointestinal cancer patients refractory to chemotherapy and the aspects related to the economic cost and compliance of the octreotide treatment.

Randomisation using cards from a computer-generated list in sealed envelopes was performed by a person not involved with the care or evaluation of the patients. The primary outcome variable in this study was the survival length. Outcome variable of secondary importance was response rate. Survival was calculated from the date of randomisation using the Kaplan–Meier estimates. The log-rank test was used to assess the difference between survival curves.

The chi-square test was used to analyse the statistical significance of the difference in clinical responses (Glantz, 1992).

### Results

### *Patients*

Between January 1990 and December 1992, 107 patients with advanced gastrointestinal carcinoma were included in the study. Patient characteristics are summarised in Table I. Fifty-five patients received octreotide and 52 the best supportive care only. In the octreotide arm primary tumours were: stomach 15, pancreas 16, colon–rectum 24; in the control group, stomach 14, pancreas 16, colon–rectum 22. Previously chemotherapeutic regimens were: for gastric cancer a weekly administration of 5-fluorouracil (5-FU), 6S-leucovorin, cisplatin and epidoxorubicin; for pancreatic cancer a weekly administration of 5FU, leucovorin and interferon alpha-2b 3 MU three times a week; for colorectal cancer a 5 day schedule of 5FU, leucovorin and interferon alpha-2b, with cycles repeated every 3 weeks.

Median time to progression from onset of chemotherapy was 6 months in the octreotide arm and 5 months in the control group (Table I). In the octreotide arm 12 patients responded to previous chemotherapy *vs* 13 in the control group. Both arms were well balanced also for other possible prognostic factors, such as performance status, liver function tests and sites of metastases.

**Table I** Patient characteristics

| | *Octreotide* | *Controls* |
|---|---|---|
| Evaluable patients | 55 | 52 |
| Sex | | |
| Male | 35 | 30 |
| Female | 20 | 22 |
| Age | | |
| Median | 68 | 66 |
| Range | 39–71 | 44–72 |
| Performance status[a] | | |
| 0 | 3 | 4 |
| I | 30 | 30 |
| II | 22 | 18 |
| Primary tumor | | |
| Stomach | 15 | 14 |
| Pancreas | 16 | 16 |
| Colon–rectum | 24 | 22 |
| Sites of metastases | | |
| Liver | 28 | 23 |
| Lung | 10 | 11 |
| Peritoneum | 19 | 16 |
| Lymph node | 8 | 9 |
| Bone | 3 | 1 |
| Response to chemotherapy | | |
| Complete response | 1 | 0 |
| Partial response | 11 | 13 |
| Stable disease | 12 | 20 |
| Progressive disease | 31 | 19 |
| Median time to progression from chemotherapy onset (months) | 6 (2–9) | 5 (2–10) |
| Stomach | 5 | 5 |
| Pancreas | 2 | 2 |
| Colon–rectum | 6 | 6 |
| Biochemical tests (median) | 480 | 470 |
| LDH (230–460 U $l^{-1}$) | (260–805) | (270–920) |
| Alkaline phosphatase (91–258) | 235 | 270 |
| | (172–470) | (135–510) |
| AST (2–31 U $l^{-1}$) | 54 | 58 |
| | (45–61) | (38–62) |
| Albumin (3–4.5 g $dl^{-1}$) | 3.1 | 3.0 |
| | (2.7–4.1) | (2.5–3.6) |

[a]ECOG performance status.

*Octreotide therapy*

The mean duration of octreotide therapy was 12 weeks (range 6–32 weeks). Four gastric cancer patients received octreotide for only 6 weeks because of a severe impairment of general conditions owing to rapid disease progression. They died after only 2 weeks. For the same reason a pancreatic cancer patient received octreotide for only 7 weeks. This thrice-daily dosing administered subcutaneously was generally well tolerated. Only five patients suffered from pain at injection sites, but it did not determine the refusal of treatment or reduce their compliance in taking the drug.

*Supportive care*

Ten patients in the octreotide arm and nine in the placebo arm required haemotransfusions. In two patients in the placebo arm a percutaneous transhepatic biliary drain was place to reduce hyperbilirubinaemia. No patient in either arm developed infections requiring hospitalisation. Antibiotic treatment was given in two patients in the octreotide arm (for pneumonia and urinary infection) and in two patients in the control group (pneumonia). Corticosteroids (methylprednisolone 8–24 mg $day^{-1}$) were administered orally as adjuvant analgesic drugs in three patients with tumour infiltration of lumbosacral plexus, and to produce increased appetite and weight gain in another three patients in the octreotide arm, while in the placebo group prednisone, at the dose of 25 mg $day^{-1}$, was given to four patients with weakness and anorexia to obtain a sense of well-being. Radiotherapy was used in four patients in the octreotide arm (two bone metastases and two symptomatic pelvic recurrence) and in six patients in the control group (one bone metastasis and five pelvic recurrences).

*Response*

No patient achieved an objective response. Twenty-five of 55 patients (45%) given octreotide (seven stomach, seven pancreas, 11 colon–rectum) showed stable disease *vs* only 8 of 52 (15%) in the control group (three somach, two pancreas, three colon–rectum) ($P<0.001$).

Patients treated with octreotide had a significant advantage in survival duration with a median survival time of 20 weeks *vs* 11 weeks in the control group. This advantage in survival was present analysing either all the patients or considering each primary tumour separately (Figures 1–4).

Twenty-two patients (40%) treated with octreotide showed relief of pain, with concomitant discontinuation in analgesic treatment requirements.

**Figure 1** Survival curve of patients treated with octreotide (■, $n=55$) *vs* controls (□, $n=52$). The difference in survival was significant by the Mantel–Cox (log-rank) test, $P<0.001$.

*Toxicity*

No severe toxicity was recorded in the octreotide arm requiring the discontinuation of octreotide. Twenty patients had asymptomatic hyperglycaemia and ten mild steatorrhoea. Three patients had abdominal cramps which disappeared spontaneously after a few days of continued therapy.

**Figure 2** Survival curves comparing patients with stomach cancer treated with octreotide ($n=15$) or not ($n=14$). There is statistical difference between the two curves: Mantel–Cox (log-rank), $P=0.003$.

**Figure 3** Survival curves comparing patients with pancreatic cancer treated with octreotide (■, $n=16$) or not (□, $n=16$). There is statistical difference between the two curves: Mantel–Cox (log-rank), $P=0.001$.

**Figure 4** Survival curves comparing patients with colorectal cancer treated with octreotide (■, $n=24$) or not (□, $n=22$). There is statistical difference between the two arms: Mantel–Cox (log-rank), $P=0.001$.

### Discussion

Somatostatin plays an important modulatory role in the secretion and growth-promoting functions of several gastrointestinal hormones that have been shown to stimulate the growth of tumour arising in the gastrointestinal tract (Townsend *et al.*, 1987).

The exact mechanism of action of somatostatin is not clear. It may influence tumour growth directly at the intracellular level, by interactions with membrane receptors, or indirectly through suppression of hormones or tumour growth factors (Reichlin, 1983).

Somatostatin receptors have been shown to be present on normal and tumoral gastrointestinal mucosa. Gastric, pancreatic and colorectal cancers have been demonstrated to possess high-affinity somatostatin receptors (Reyl & Lewin, 1982; Reyl-Desmars and Lewin, 1982; Dy *et al.*, 1992).

There are, however, other mechanisms that are likely to be involved, such as inhibition of the secretion of growth hormone and insulin, and direct inhibition of insulin-like growth factor (IGF-I), IGF-II and other growth factors that have been recently shown to be potent stimulators of gastrointestinal tumour cell proliferation (Durrant *et al.*, 1991; Waston *et al.*, 1992).

Other studies have suggested that somatostatin can suppress the release or action of the gastrointestinal hormones gastrin, cholecystokinin (CCK) and secretin. This is a particularly attractive hypothesis, because gastrin and CCK are important trophic hormones for gastrointestinal mucosal cells. This could partly explain the anti-cancer effect of somatostatin (Harty *et al.*, 1985; Schally *et al.*, 1986; Charnley *et al.*, 1989; Karnik *et al.*, 1989; Watson *et al.*, 1989).

Another possible mechanism by which somatostatin might inhibit tumour growth is interference with the synthesis of autocrine growth factors by tumour cells. This action of somatostatin might involve the inhibition of not only endocrine but also paracrine and autocrine growth factors (Goustin *et al.*, 1986; Lippman *et al.*, 1986). Somatostatin could also inhibit oncogene products, several of which are similar to growth factors or their receptors (Doolittle *et al.*, 1983; Downward *et al.*, 1984). Consequently, somatostatin could be of value for impeding the growth of cancers, such as gastric, pancreatic and colorectal, in which gastrointestinal hormones as well as growth factors might be involved (Schally *et al.*, 1987). However, the half-life of somatostatin in plasma (estimated to be 1.1–3.0 min in humans) presents difficulties for its clinical use (Sheppard *et al.*, 1979). For this reason, several somatostatin analogues have been developed. Octreotide has a longer half-life (90 min) and a duration of action of about 8 h after subcutaneous injection (Bauer *et al.*, 1982). It is three times more potent *in vitro* and up to 70 times more active *in vivo* than native somatostatin.

Several studies have demonstrated that octreotide is able to inhibit *in vitro* or *in vivo* growth of gastrointestinal tumours (Reyl and Lewin, 1982; Townsend *et al.*, 1987; Schally, 1988).

On the basis of experimental data, pilot clinical trials with octreotide were carried out in gastrointestinal tumours. However, in spite of the intriguing preclinical data, initial preliminary studies led to disappointing results.

Klijn *et al.* (1990) treated 34 patients with gastrointestinal cancers, obtaining 27% stable disease, but survival remained discouraging. However, it was of interest that most patients experienced subjective improvement in the absence of serious side-effects. Savage *et al.* (1987) treated ten patients (four pancreatic cancers, four colorectal cancers, two gastric cancers) without finding any indication that octreotide can alter the rate of growth of advanced gastrointestinal tumours.

A recent trial evaluating octreotide (150 μg thrice daily, subcutaneously) *vs* placebo in 260 advanced chemonaive colorectal cancer patients had showing no difference in terms of time to progression and survival between the two arms suggested that octreotide, at least in this dose and schedule, is ineffective in advanced colorectal cancer (Krook *et al.*, 1993). However, in this study, published until now only in the form of a meeting abstract, some important aspects of outcome, such as the duration of octreotide therapy, the further treatment received by patients with progressive disease after octreotide and the supportive care practised in patients becoming symptomatic during the course of disease, are not available, thus limiting, in our opinion, a complete interpretation of these data.

The only partially positive study was presented by Smith *et al.* (1992), who found a modest increase in survival in 12 colorectal cancer patients treated with octreotide, but no objective responses.

These conflicting results probably reflect the difficulty in assessing the activity of an agent such as octreotide and the lack of knowledge of its proper dose and scheme of administration. In fact, apart from the study of Krook *et al.* (1993), previous pilot studies were performed to evaluate the clinical activity of octreotide in terms of objective responses (Savage *et al.*, 1987; Klijn *et al.*, 1990; Smith *et al.*, 1992). However, it is possible that treatment with octreotide or other similar agents could be valuable if it consistently results in stabilisation of disease, and if this is associated with an improvement in survival.

A second problem arising from the studies cited above is the octreotide dose, which was probably too low to achieve an anti-tumour effect. In fact, experimental data suggest that a serum octreotide level of about 1200 ng ml$^{-1}$, which is equivalent to $1.2 \times 10^{-6}$ M, appears to be effective (Dy *et al.*, 1992). This concentration can be achieved clinically after a subcutaneous injection of at least 200 μg of octreotide repeated every 8 h. (Del Pozo *et al.*, 1988). On the contrary, octreotide was generally administered at a dose of 200 μg twice daily or 100–150 μg thrice daily; this corresponds to 60–75% of the optimal dose as judged from pharmacokinetic models (Del Pozo *et al.*, 1986; Dy *et al.*, 1992).

Another critical point in previous studies could be the administration of octreotide for a long time without discontinuation. Preclinical data have revealed that after continuous administration of octreotide desensitisation or tachyphylaxis of its inhibitory effect on receptors and plasma growth hormone, insulin and IGF-I concentrations develops within 6–10 days, but this could be reduced if 'drug-free' days were inserted in the protocol (Redding and Schally, 1983; Lamberts *et al.*, 1987).

These experimental data suggest that the discouraging results obtained until now could be caused by the use of a suboptimal schedule.

Our study, employing subcutaneous octreotide 200 μg three times a day for 5 days a week, does suggest an advantage for octreotide therapy in gastrointestinal cancer patients. We observed a doubling of survival time in treated patients with respect to the control group, considering either all the patients or each primary tumour separately. Nevertheless, we are conscious that the interpretation of our results requires caution, particularly in view of the negative randomised NCCGT trial in untreated colorectal cancer patients (Krook *et al.*, 1993). Although in our study both groups of patients were looked after by the same medical and nursing staff, and factors predicting for outcome, such as performance status, sites of metastases, response to previous chemotherapy and adequate organ functions were well balanced in the two arms, the possible confounding effects of different terminal care practices in the two arms and the lack of a double-blind design cannot exclude completely a degree of bias in the conduct of the study. It is of note that, as in other studies, 40% of patients in the octreotide arm showed subjective improvement with relief of pain and discontinuation of analgesic therapy. This suggests that quality of life could be improved by octreotide therapy. Unfortunately, we had not included a quality of life assessment in this trial. Further studies, especially in a population such as this, should consider some sort of quality of life measurements.

In conclusion, although our results are encouraging, we think that additional studies need to confirm these favourable data and to clarify other important questions, e.g. the relationship between somatostatin receptors status and response, the optimal dose and timing of octreotide administration and, last but not least, the impact of octreotide treatment in terms of not only survival but also patients' quality of life.

### References

AHLGREN JD AND MACDONALD JS. (1992). *Gastrointestinal Oncology*. J.B. Lippincott: Philadelphia.

BAUER W, BRINER U, DOEFNER W, HALLER R, HUGUENIN R, MARBACH P, PETCHER TJ AND PLESS J. (1982). SMS 201-995: a very potent and selective octapeptide analogue of somatostatin with prolonged action. *Life Sci.*, **31**, 1133–1140.

BODEN G, RYAN IG, EISENSCHMIDT BL, SHELMET JJ AND OWEN OE. (1986). Treatment of inoperable glucagonoma with the long acting somatostatin analog SMS 201-995. *N. Engl. J. Med.*, **314**, 1686–1689.

CHANG RSL, LOTTI VJ, CHEN TB AND KUNKEL KA. (1986). Characterization of the binding of [3H]-(+)-L-364, 718: a new potent, nonpeptide cholecystokinin antagonist radioligand selective for peripheral receptors. *Mol. Pharmacol.*, **30**, 212–217.

CHARNLEY RM, THOMAS WM, STANLEY J AND MORRIS DL. (1989). Serum gastrin concentrations are higher in colorectal cancer patients. *Gut*, **30**, 712–713.

DEL POZO E, NEUFELD M, SCHLUTER K, TORTOSA F AND CLARENBACH P. (1986). Endocrine profile of a long-acting somatostatin derivative SMS 2201-995: study in normal volunteers following subcutaneous administration. *Acta Endocrinol.*, **111**, 433–439.

DOOLITTLE RF, HUNKAPILLER MW, HOOD LE, DEWARE SC, ROBBINS KC AND AARONSON SA. (1983). Simian sarcoma virus one gene r-sis is derived from the gene encoding a platelet derived growth factor. *Science*, **221**, 275–277.

DOWNWARD J, YARDEN Y, MAYES E, SCRAGE G, TOTTY N AND STOCKWELL P. (1984). Close similarity of epidermal growth factor receptor and r-erb B oncogene protein sequences. *Nature*, **307**, 521–527.

DURRANT LG, WATSON SA, HALL A AND MORRIS DL. (1991). Costimulation of gastrointestinal tumour cell growth by gastrin, transforming growth factor alpha and insulin like growth factor-I. *Br. J. Cancer*, **63**, 67–70.

DY DY, WHITEHEAD RH AND MORRIS DL. (1992). SMS 201.995 inhibits in vitro and in vivo growth of human colon cancer. *Cancer Res.*, **52**, 917–923.

GLANTZ SA. (1992). *Primer of Biostatistics*. McGraw Hill: New York.

GOUSTIN AS, LEOF EB, SHIPLEY GS AND MOSES HC. (1986). Growth factors and cancer. *Cancer Res.*, **46**, 1015–1029.

HARTY RF, MAICO DG AND MCGUIGAN JE. (1985). Postreceptors inhibition of antral gastrin release by somatostatin. *Gastroenterology*, **88**, 675–680.

HUDD C, LAREGINA MC AND DEVINE JE. (1989). Response to exogenous cholecystokinin and six human gastrointestinal cancers xenografted in nude mice. *Am. J. Surg.*, **157**, 386–394.

KARNIK PS, MONAHAN SJ AND WOLFE MM. (1988). Inhibition of gastrin gene expression by somatostatin. *J. Clin. Invest.*, **83**, 367–372.

KLIJN JGM, HOFF AM, PLANTING ASTH, VERWEIJ J, KOK T, LAMBERTS SWJ, PORTENGEN H AND FOEKENS JA. (1990). Treatment of patients with metastatic pancreatic and gastrointestinal tumours with the somatostatin analogue Sandostatin: a phase II study including endocrine effects. *Br. J. Cancer*, **62**, 627–630.

KOBORY O, VUILLOT MT AND MARTIN F. (1982). Growth responses of rat stomach cancer cells to gastro-enteropancreatic hormones. *Int. J. Cancer*, **30**, 65–67.

KRAENZLIN ME, CHANG JLC, WOOD SM, CARR DH AND BLOOM SR. (1985). Long term treatment of VIPoma with somatostatin analogue resulting in remission of symptoms and possible shrinkage of metastases. *Gastroenterology*, **88**, 185–187.

KROOK J, GOLDBERG RM, MOERTEL CG AND WIEAND HS. (1993). A phase III evaluation of the somatostatin analogue octreotide in the therapy of asymptomatic advanced colon cancer: a North Central Cancer Treatment Group study (NCCTG). *Proc. Am. Soc. Clin. Oncol.*, **12**, 191.

KVOLS LK, BUCK M, MOERTEL CG, SCHUTT AJ, RUBIN J, O'CONNEL MJ AND HAHN RG. (1987). Treatment of metastatic islet cell carcinoma with somatostatin analogue (SMS 201-995). *Ann. Intern. Med.*, **107**, 162–168.

LAMBERTS SWJ, KOPER JW AND REUBI JC. (1987). Potential role of somatostatin analogues in the treatment of cancer. *Eur. J. Clin. Invest.*, **17**, 281–287.

LIPPMAN ME, DICKSON RB, KASID A, GELMANN EE, DAVIDSON N AND MCMANAWAY M. (1986). Autocrine and paracrine growth regulators of human breast cancer. *J. Steroid. Biochem.*, **24**, 147–154.

MILLER AB, HOODGSTRATEN B, STAQUET M AND WINKLER A. (1981). Reporting results of cancer treatment. *Cancer*, **47**, 207–214.

REDDING TW AND SCHALLY AV. (1983). Inhibition of growth of pancreatic carcinomas in animal models by analogs of hypothalamic hormones. *Proc. Natl Acad. Sci. USA*, **80**, 3485–3488.

REICHLIN S. (1983). Somatostatin. *N. Engl. J. Med.*, **309**, 1495–1501.

REYL FJ AND LEWIN MJM. (1982). Intracellular receptor for somatostatin in gastric mucosal cells: decomposition and reconstitution of somatostatin-stimulated phosphoprotein phosphatases. *Proc. Natl Acad. Sci. USA*, **76**, 978–982.

REYL-DESMARS F AND LEWIN MJM. (1982). Evidence for an intracellular somatostatin receptor in pancreas: a comparative study with reference to gastric mucosa. *Biochem. Biophys. Res. Commun.*, **46**, 1324–1331.

SALTZ L, TROCHANOWSKY MPA, BURKLEY M, HEFFERNAN B, NIEDZWIECKI D, TAO Y AND KELSEN D. (1993). Octreotide as an antineoplastic agent in the treatment of functional and nonfunctional neuroendocrine tumors. *Cancer*, **72**, 244–248.

SAVAGE AP, CALAM J, WOOD CB AND BLOOM SR. (1987). SMS 201.995 treatment and advanced intestinal cancer: a pilot study. *Aliment. Pharmacol. Ther.*, **1**, 133–139.

SCHALLY AV. (1988). Oncological applications of somatostatin analogues. *Cancer Res.*, **48**, 6977–6985.

SCHALLY AV, CAI RZ, TORRES-ALERAN I, REDDING TW, SZOKE B, HIEROWSKI MT, COLALUCA J AND KONTUREK S. (1986). Endocrine, gastrointestinal and antitumor activity of somatostatin analogue. In *Neural and Endocrine Peptides and Receptors*. Moody TW. (ed.) pp. 73–83. Plenum Publishing: New York.

SCHALLY AV, REDDING TW, CAI RZ, PAZ JI, BEN-DAVIS M AND COMARU-SCHALLEY AM. (1987). Somatostatin analogs in the treatment of various experimental tumors. In *International Symposium on Hormonal Manipulation of Cancer Peptides, Growth Factors and New (anti)steroidal agents*. Klijn JGM. (ed.) pp. 431–440. Raven Press: New York.

SHEPPARD MC, SHAPIRO B, PIMSTONE B, KRONHEIN S, BERELOWITZ M AND GREGORY M. (1979). Metabolic clearance and plasma half disappearance time of exogenous somatostatin in man. *J. Clin. Endrocrinol. Metab.*, **48**, 50–53.

SINGH P, WALKER JP, TOWNSEND CM AND THOMPSON JC. (1986). Role of gastrin and gastrin receptors on the growth of a transplantable mouse colon carcinoma (MC-26) in BALB/c mice. *Cancer Res.*, **46**, 1612–1616.

SMITH JP, CROITORU R, DOLL D, THORNTON C AND PERRY MCC. (1992). Effects of octreotide, a long-acting somatostatin analog on advanced colon cancer. *Gastroenterology*, **102** (Suppl.), 399.

TOWNSEND CM, SINGH P AND THOMPSON JC. (1987). Possible role of gut hormones in cancer. In *Gastrointestinal Endocrinology*, Thompson JC. (ed.) pp. 178–183. McGraw-Hill: New York.

TOWNSEND CM, SINGH P AND THOMPSON JC. (1989). Effects of gastrointestinal peptides on gastrointestinal cancer growth. *Gastroenterol. Clin. N. Am.*, **18**, 777–791.

WATSON SA, DURRANT LG, CROSBIE JD AND MORRIS DL. (1989). The in vitro growth response of primary human colorectal and gastric cancer cells to gastrin. *Int. J. Cancer*, **43**, 692–696.

WATSON SA, MORRIS DL, DURRANT LG, ROBERTSON JF AND HARDCASTLE JD. (1992). Inhibition of gastrin-stimulated growth of gastrointestinal tumour cells by octreotide and the gastrin/cholecystokinin receptor antagonists, proglumide and lorglumide. *Eur. J. Cancer*, **8**, 1462–1467.